# Piloting a Telephone Based Health Coaching Program for Pregnant Women: A Mixed Methods Study

**DOI:** 10.1007/s10995-019-02735-2

**Published:** 2019-02-12

**Authors:** Chris Rissel, Santosh Khanal, Jane Raymond, Vanessa Clements, Kit Leung, Michael Nicholl

**Affiliations:** 1 NSW Office of Preventive Health, Liverpool, NSW Australia; 2 NSW Ministry of Health, North Sydney, NSW Australia; 3 Department of Obstetrics and Gynaecology, Royal North Shore Hospital, St Leonards, NSW Australia

**Keywords:** Health promotion, Health coaching, Gestational weight gain, Obesity, Maternal obesity

## Abstract

**Abstract:**

*Objectives* Get Healthy in Pregnancy (GHiP) is a telephone based lifestyle coaching service for pregnant women, in New South Wales, Australia. GHiP had two service options; a telephone-based health coaching program consisting of up to 10 calls and information only (including one call). This study sought to compare the outcomes of the two GHiP options, to determine the characteristics of women likely to use the service and to explore the feedback from women and health professionals. *Methods* A pragmatic stratified clustered randomised controlled trial was conducted. Two metro and three rural hospitals were randomised into health coaching or information only arms. Self-reported measures of height and weight and health behaviours (dietary and physical activity) were collected at baseline and 36 weeks gestation. Process evaluation included descriptive analysis of routine program data, and semi-structured interviews with participants and health professionals. *Results* Of 3736 women screened, 1589 (42.5%) were eligible to participate, and of those eligible, 923 (58.1%) were recruited. More women in the health coaching arm gained weight within the target range for their BMI at 36 weeks gestation (42.9%) compared with information only (31.9%). Women found GHiP to be useful and supportive and midwives and doctors said that it facilitated conversations about weight with pregnant women. *Conclusions for Practice* Telephone-based lifestyle programs integrated with routine clinical care show promise in helping pregnant women achieve healthy gestational weight gain, but in this case was not significantly different from one information telephone call. Strong positive feedback suggests that scaled-up service delivery would be well received.

**Trial Registration:**

ACTRN12615000397516 (retrospectively registered).

## Significance Statement

*What is already known on this subject?* Gestational weight gain for most women exceeds recommendations, and can lead to adverse health outcomes for women and their children. Interventions to support healthy weight gain are needed. *What this study adds?* Telephone coaching to support pregnant women to achieve healthy weight gain is well received by the women and supports health professionals in a professional dialogue about healthy weight. A series of telephone coaching calls led to a non-significantly higher proportion of women achieving their target weight gain compared to information only and one telephone call.

## Background

A large body of evidence links excessive gestational weight gain (EGWG) with poor maternal and infant health outcomes (Gaillard et al. 2013; Mamun et al. 2010; Stotland et al. 2004), leading to an increased likelihood of postpartum obesity in both women (Mamun et al. 2010) and their children (Ludwig & Currie). In turn, these early adverse health outcomes lead to an increased risk of chronic disease later in life (Muktabhant et al. 2012). The Institute of Medicine (IOM) (Institute of Medicine (US) and National Research Council (US), 2009) has recommended a range for healthy weight gain for specific pre-pregnancy BMI categories based on the least risk of adverse perinatal outcomes (Stotland et al. 2006).

Gestational weight gain for most women exceeds the IOM recommendations (Institute of Medicine (US) and National Research Council (US), 2009), and in Australia, the prevalence of EGWG has been reported to be between 38% and 67% (Chung et al. 2013; de Jersey et al. 2012). The first national guidelines for antenatal care in Australia published in 2012 recommended that clinicians give women advice about appropriate weight gain during pregnancy in relation to their pre-pregnancy BMI (Australian Health Ministers’ Advisory Council 2012). Women should receive interventions related to weight gain as early as possible in their pregnancy to help them make healthy choices that will maximise health outcomes for them and their babies.

A number of interventions (Thangaratinam et al. 2012) targeting weight gain in pregnancy have been developed but the literature reports that passive interventions such as leaflets and brochures which have limited effectiveness, or they have a face-to-face component which make them costly and resource-heavy when implemented on a large scale. A meta-analysis with 44 studies found that overall, the intervention group had a 1.42 kg reduction in gestational weight gain with any intervention compared with the control group, and with dietary intervention resulting in the largest reduction in maternal gestational weight gain (3.84 kg), and with improved pregnancy outcomes compared with other interventions (Thangaratinam et al. 2012). The quality of the studies varied considerably, with the quality of those studies addressing gestational weight gain considered moderate (Thangaratinam et al. 2012). To contribute to outcomes at a population level, the interventions need to be implemented widely and with sufficient intensity to improve individual risk behaviours (Cahalin et al. 2015). These outcomes are not achievable with most of the currently available interventions.

One potential intervention for promoting healthy weight gain during pregnancy is telephone-based health coaching. Telephone-based health coaching delivered by qualified health professionals unrelated to the client’s clinical care is a well-established care model for patients with chronic disease (Goode et al. 2012). Health coaching is designed to support clients through regular contact to achieve a healthier lifestyle through the use of behaviour change theory, and thereby also supporting the clinicians to support the overall health of their patients. An example is the Get Healthy Information and Coaching Service (GHS), which is a free telephone-based health coaching service that has been available to all people over 18 years of age in New South Wales (NSW), Australia, since 2009. People using this service can opt to receive information only or enrol in a 6-month coaching program during which they receive 10 individually tailored calls from university qualified coaches. The coaching calls aim to support participants in making sustained improvements in healthy eating, physical activity and achieving or maintaining a healthy weight. Participants who complete the 6-month coaching program have been shown to lose an average of 3.9 kg and reduce their waist circumference by 5.0 cm (O’Hara et al. 2012).

Using the GHS model, we developed the Get Healthy in Pregnancy (GHiP) service (Clements et al. 2016), with two service delivery options (6-months of health coaching and one call for information only). In this paper we compare the outcomes of the information only and telephone based coaching options of the GHiP service, determine the characteristics of pregnant women most likely to utilise the program and explored the feedback from women and health professionals related to usefulness of GHiP and program uptake and retention.

## Materials and Methods

### Study Design

This study employed a pragmatic stratified cluster randomised design, with stratification of participants by healthy weight and overweight/obese pre-pregnancy BMI and of hospitals by metropolitan and rural regions. Hospitals were randomised into the two GHiP options; telephone-based health coaching and information only. Given the evidence supporting appropriate weight gain during pregnancy for the health of the woman and her baby, a pure control group was not considered ethically acceptable.

Process evaluation was conducted which included an audit of the women who agreed to participate in the study but did not enrol into the program, and semi-structured interviews with women, midwives and the medical staff at the participating hospitals. The service provider that delivered the program collected routine program data including and the status of each participant i.e. continuing or withdrawn, the number of calls received by participants and the duration of each call.

### Study Setting

The study was conducted in the antenatal clinics of five NSW public hospitals that account for approximately 10% of the total births in NSW. Three of the hospitals were rural (Orange Base, Lismore Base and Dubbo Base) and two were metropolitan (Liverpool and Blacktown). Two of the rural hospitals (Orange and Dubbo) were combined into one unit for randomisation because they are in the same local health district and their aggregated total number of births in 2012 was similar to the total of the other rural hospital in the study.

### Ethics and Consent

The study was approved by the Human Research Ethics Committee of South Western Sydney Local Health District (HREC/14/LPOOL/131) and site specific approvals were obtained for each study site. Informed consent was obtained from the women by their attending midwives prior to the inclusion of the women in the study.

### Inclusion and Exclusion Criteria

The inclusion criteria for the study were English speaking, 18 years and over, singleton pregnancy and gestation of 18 weeks or under. Women with medical conditions that may have impacted on their ability to participate in the study were identified to ensure safety to participate in the study. These women were either excluded from the study or were required to receive medical clearance prior to their inclusion (Clements et al. 2016). Women who were underweight at pre-pregnancy were excluded from the study.

### Power Analysis/Sample Size

A total of 167 women were required in each of the health coaching and the information only arms arm to detect a 15% difference in the proportion of participants who gained weight within the target weight for their pre pregnancy BMI at a significance level of 0.05 with a power of 80%. We aimed to recruit 280 women in the health coaching arm allowing for an attrition rate of 60% and 235 women in the information only arm allowing for an attrition rate of 30%.

### Recruitment Procedures

During the recruitment period (Sept 2014–Sept 2015) midwives screened every woman presenting for their first antenatal appointment to determine eligibility to be enrolled in the study. Women were screened for their eligibility and invited to participate in the study by the midwives during the women’s first antenatal appointment at the hospital. The women self-reported their pre-pregnancy height and weight to the midwives.

### Intervention Group Procedures

In the *information only arm*, midwives discussed gestational weight gain recommendations with the women and referred them to the GHiP service. When the women enrolled into the GHiP service, they were provided with one-off 20–30 min information only telephone call during which the women were provided information about appropriate weight gain in pregnancy and baseline data was collected. Evidence based written resources regarding weight gain in pregnancy were sent to the participants by post. The resources included factsheets about healthy eating, physical activity and weight gain during pregnancy and an information booklet about activities that could be undertaken to be healthy.

In the *telephone-based health coaching arm*, women received identical evidence-based written resources plus a journey booklet to record their progress and up to 10 health coaching calls (8 during pregnancy, 2 post-pregnancy). The journey booklet was designed as a resource for the women to track their progress through the program and was not used as one of the study evaluation tools. Baseline data was collected during the first call. The topics discussed during the calls are listed in Table 1.

**Table 1:** Gestation specific topic guide for the health coaches to tailor their conversations with the participants

Call No.	Stage of pregnancy	Content
1	18–21 weeks of gestation	• Baseline data collection
1–6	1st and 2nd trimester	• Common problems of pregnancy, why they occur and what might help • Common discomforts and what to do to relieve them (e.g. for nausea, have a snack and avoid greasy/spicy foods) • Healthy eating: Australian Guide to Healthy Eating (5 food groups), snack ideas, myths (e.g. “eating for two”) and recommended weight gain during pregnancy, important nutrients during pregnancy and lactation (e.g. folate, iodine, iron), foods to avoid • Physical activity: benefits of exercise, tips and precautions, stretching exercises, type, intensity, frequency and duration, importance of variety, incidental daily activity
7–8	3rd trimester	• Feeding your baby and benefits of breastfeeding • Healthy eating: portion sizes and serves, healthy plate, keeping motivated, food labels • Physical activity: posture, back care and symptom cycle, pelvic floor exercises, physical activity towards the end of pregnancy
9–10	Post natal	• Healthy eating: eating out, tips for busy lives and easy meals • Physical activity: relaxation • Behaviour maintenance

Both GHiP arms commenced between 12 and 22 weeks gestation and all the phone calls were made by university qualified health professionals such as dieticians and exercise physiologists. Post study data was collected at 36 weeks gestation for both arms.

### Data Collection

Data for the study was collected at screening (by midwives in antenatal clinics), pre-program (by health coaches during enrolment into GHiP for health coaching arm and during one-off information only call for information only participants) and at 36 weeks gestation (by health coaches or research assistant). The screening data, collected by the midwives at the hospital, included information regarding the inclusion and exclusion criteria for the study and the women’s height and current weight. The women self-reported their pre-pregnancy weight in line with current routine maternity data collection across NSW. The pre-program and 36 week data collection included self-reported weight and validated self-reported dietary (Barr et al. 2008; McLennan and Podger 1998; Wright and Scott 2000), and physical activity measures (Australian Institute of Health and Welfare 2003; Smith et al. 2005). The items collected in the dietary questionnaire include fruit and vegetable serves per day, cups of soft drinks per day and number of take away meals per week. Similarly the physical activity questionnaire included questions about walking and moderate and vigorous physical activity.

Semi-structured interviews with the women were conducted with a purposive sample of study participants at various stages of the program. Women were selected on the basis of their BMI status, the number of calls they received (0 calls, 1–3 calls completed, 4–7 calls completed and the 36 week call completed) and whether they were from metro or rural regions. The health professional semi-structured interviews were conducted with a convenient sample of midwives (N = 19) and medical practitioners (N = 5).

### Data Analysis

A logistic regression of the combined screening and baseline data was conducted to determine the women who would be most likely to agree to participate in the study and enroll into the program. For the women who completed the 36 weeks gestation data collection, the differences between the two arms in mean weight change and changes in dietary scores at 36 weeks gestation and the proportions of pregnant women who achieved weight gain within the recommended range for their pre pregnancy BMI were conducted descriptively. All statistical analyses were done using IBM SPSS statistics v21 (IBM Inc, NY, USA). We followed the COREQ criteria for reporting qualitative research.

In this paper, we explored the women’s participation and withdrawal from the study and the usefulness of GHiP to the midwives and the medical practitioners in the antenatal clinics. Other aspects of the process evaluation will be discussed in a subsequent paper. In the first few months of recruitment, an audit of randomly selected non-enrollees who agreed to participate in the study but did not subsequently enroll into GHiP was conducted. Semi-structured interviews were conducted with midwives, medical practitioners and with women who completed and those women who withdrew from GHiP at various stages. A thematic analysis of the interview data was conducted to identify the reasons for discontinuing from the program prior to the 36 week data collection point. The coding of the transcripts was undertaken and checked by independent coders, and themes and subthemes were extracted from the coded data. We included all of the elements of the RECORD checklist to match our study design.

## Results

**Fig. 1:**
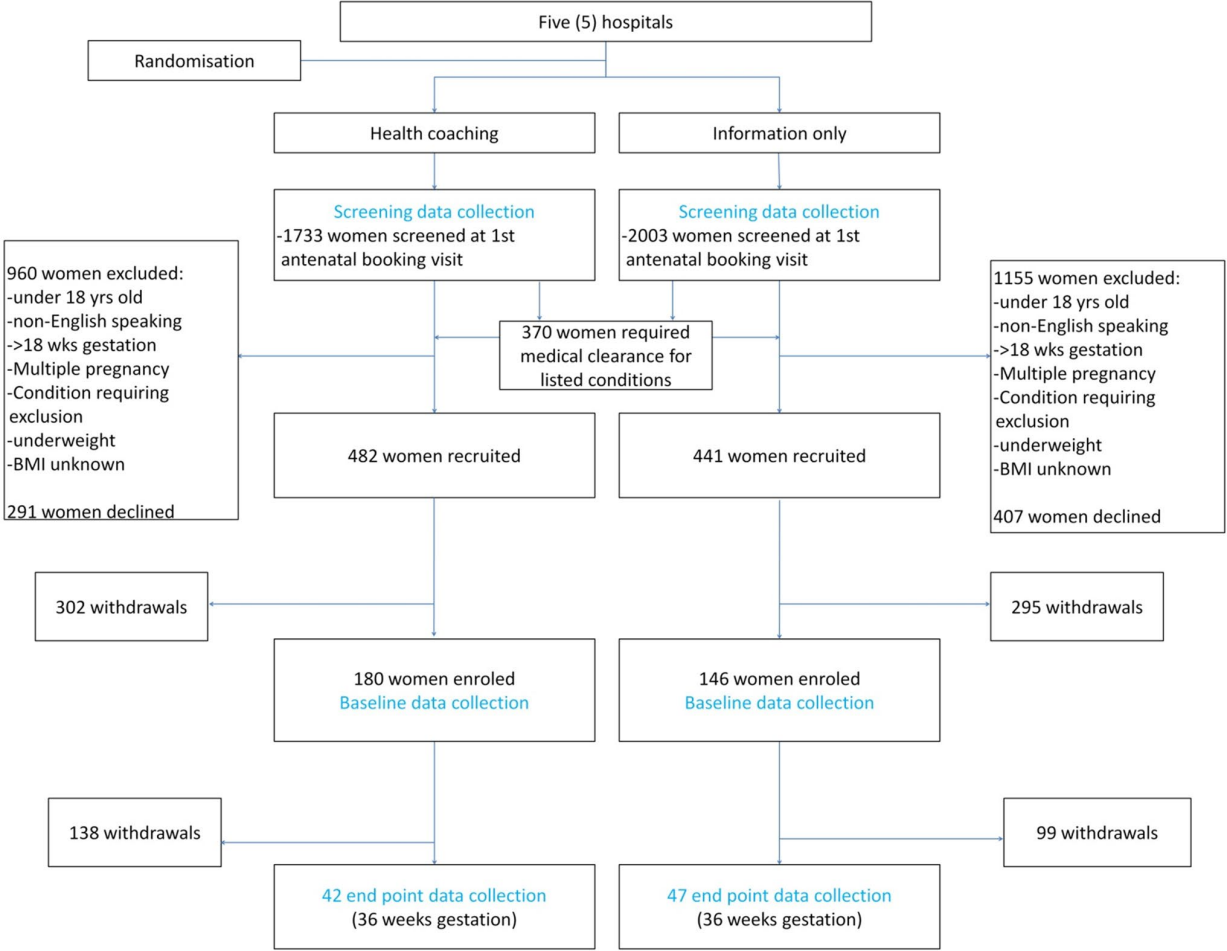
The study flowchart

Overall 3736 women were screened across all the five hospitals and 1589 (42.5%) were eligible to participate in the study. Of the eligible women, 923 (58.1%) agreed to participate and were recruited into the study. A total of 322 women (34.9% of women recruited) enrolled into the program, of whom 89 (27.6%) completed the 36 week call. No differences in the withdrawal patterns between the health coaching and the information only arms were found (Fig. 1).

In the health coaching arm, 64.3% (27/42) of the women who reached the 36 week data collection point received all of the eight pre pregnancy calls. The number of calls received by the remaining women in the health coaching arm who reached the 36 week data collection point were evenly spread out (2 calls, n = 2; 3 calls, n = 2; 4 calls, n = 4; 5 calls, n = 2, 6 calls, n = 3; 7 calls, n = 2). In the health coaching arm, the first two calls were approximately 15 min in duration and the remaining calls were on average approximately 10 min each, and the information only call in the information only arm was on average 22 min in duration.

### Program Uptake and Retention

No differences in the average age and pre pregnancy weight of women who were ineligible or eligible for the study were seen. Women attending rural hospitals were more likely to be ineligible (n = 979, 74.1%) than women attending metro hospitals (n = 1104, 45.9%) (p < 0.01). Attending the antenatal clinic after 18 weeks of gestation was the most common cause of ineligibility in both rural (n = 937, 95.7%) and metro hospitals (n = 835, 75.6%), although more so in rural hospitals (p < 0.01). Table 2 shows the proportion of women ineligible for the study by reason and study arm.

**Table 2:** Reasons for ineligibility to participate in the study

	Health coaching (N = 960, %)	Information only (N = 1155, %)	Total (N = 2115, %)
Gestational age over 18 weeks	640 (66.7)	768 (66.5)	1408 (66.6)
Non-English speaking	85 (8.9)	103 (8.9)	188 (8.9)
Multiple pregnancy	22 (22.9)	21 (18.2)	43 (20.3)
Pre-existing medical condition	36 (37.5)	25 (21.6)	61 (28.8)
Underweight	93 (9.7)	80 (6.9)	173 (8.2)
Under 18 years of age	38 (4.0)	29 (2.5)	67 (31.7)
Unknown BMI	131 (13.6)	129 (11.2)	260 (12.3)

Of the eligible women, a higher proportion of women from the metro region (n = 602, 46.2%) than rural (n = 120, 35.1%) (p < 0.01) declined to participate in the study, but there were no differences in the recruitment rates when compared by the study arms (health coaching, n = 291, 33.6%; information only, n = 407, 35.2%). The main reasons provided by the women for declining to participate in the study were non-interest in managing weight (n = 191, 27.4%) and lack of time (n = 148, 21.0%).

The logistic regression of the combined screening and baseline data showed that younger women from rural areas (p < 0.01) and women who had more previous pregnancies more than 20 weeks (p = 0.04) were more likely to enrol into the program than other women. re-Pre-pregnancy BMI or the location of hospital on the recruitment into the study had no significant effect on outcomes. However, a higher proportion of women who were overweight or obese pre pregnancy compared with women of normal BMI were more likely to enrol into the program but withdraw before 36 weeks (p < 0.01). The demographics of women screened, recruited into the study and enrolled in the GHiP service were similar in both health coaching and information only arms (Table 3).

**Table 3:** Comparison of the demographics and weight status of women who were recruited, enrolled and withdrew by intervention arm

	Health coaching				Information only			
	Screened	Recruited	Enrolled in GHS—pre-program data	Withdrew from GHS after enrolling	Screening	Recruited	Enrolled in GHS—pre-program data	Withdrew from GHS after enrolling
Age (years ± SD)	28.7 (5.7)	29.7 (6.5)	29.5 (4.7)	29.4 (4.3)	28.7 (6.3)	29.0 (5.1)	28.5 (5.2)	27.8 (5.3)
Average pre pregnancy weight (kg ± SD)	61.1 (19.3)	65.0 (19.6)	62.3 (7.0)	71.4 (25.2)	58.8 (18.4)	61.1 (18.5)	58.6 (5.8)	62.1 (17.8)
% OWO (pre pregnancy)	47.4	50.0	60.2	64.0	40.8	50.0	50.3	55.2
% From rural hospitals	37.4	31.5	41.7	38.2	33.6	17.4	20.8	23.2

### Program outcomes

The results for weight and health behaviour outcomes of the cohort of 89 women who completed 36 weeks of the program are provided in Table 4. The proportion of women who gained weight within the target range for their BMI between the health coaching (42.9%, 18/42) and the information only (31.9%, 15/47) arms, an 11.0% difference. However, this result was not statistically significant (p = 0.29). Although positive changes were seen in both arms, the reductions in daily soft drink intake and weekly takeaway consumption were slightly higher for the health coaching arm than information only, as expected.

**Table 4:** Comparison between intervention and control arms of the changes in health behaviours and outcome measures

	Health coaching (N = 42)	Information only (N = 47)
Weight gain (kg ± SD)	11.3 (± 5.5)	13.6 (± 7.7)
N (%) participants with weight gain within target range	18 (42.9)	15 (31.9)
Change in health behaviours (post–pre)		
No. times walking at least 30 min/week	0.8 (± 2.6)	1.3 (± 3.1)
No. times of moderate to vigorous PA/week	0.2 (± 1.6)	0.4 (± 1.6)
Veg serves/day	0.5 (± 1.3)	0.2 (± 1.7)
Fruit serves/day	0.0 (± 1.0)	0.0 (± 1.2)
Cups soft drinks/day	− 0.4 (± 0.9)	− 0.1(± 0.9)
Times take away/week	− 0.4 (± 1.0)	0.1 (± 1.0)

### Process evaluation

In the audit of 26 randomly selected women who agreed to participate but did not enrol, 12 (46.2%) were successfully contacted. The phone numbers were incorrect or had been disconnected for five women who could not be contacted, and 12 women did not answer their phones.

One of the key themes that emerged in the context of study recruitment was that the women agreed to participate in the study primarily because they were asked by their midwife. In some instances, women also pointed out their interest in maintaining weight during and after pregnancy as their reasons for participation.

‘I just thought I’ll do it, because the midwife spoke to me about it and I just agreed to it.’ (B5_P)

‘Cause I was over the weight I wanted to be when I fell pregnant anyway. So I just wanted to make sure that I didn’t put on ridiculous amounts of weight, that I’d have to then try and lose, once I had bub.’ (C2_P)

The reasons consistently provided by the women for declining to participate in the study (audit of non-enrollees) or withdrawing after enrolling (semi-structured interviews) were lack of time and non-interest in weight management, healthy eating or exercise during pregnancy. Phone related issues such as disconnected phone numbers and missed or no calls were also provided by the women as reasons for non-enrolment or non-continuation.

“I found it difficult to keep up with the phone calls. I also have a three-years-old daughter as well, and just managing my time, and I was still working and all that, sort of, thing, so it was a bit difficult. So I just kept missing the phone calls.” (D8_P)

The midwives and medical practitioners were generally positive about the program and suggested that GHiP facilitated their conversations about weight with women and helped them frame the topic positively as a routine conversation they had with all women, not just with women who were overweight or obese.

“Actually it’s been good, because I can offer them something for it, so it’s not just look you’re overweight, you need to do something about it, it’s this is something that can help you through it.” (Y12_R)

“It’s been helpful because it [the service] gives another way of communicating with the patients and approaching them with their weight gain—it gives more support for what we are talking about in the clinic.”(X6_DR)

## Discussion

Our study was designed to examine the impact of GHiP service, however, the results have highlighted the issues with reach and uptake of a telephone based health coaching service for pregnant women. The findings suggest that the service was of some value for the women who completed it in helping them gain weight within the target range for their BMI but the inadequate sample size and low retention rates make it difficult to assure the validity of the outcomes. Also, GHiP facilitated a conversation between the health practitioners and the pregnant women about weight, a topic often considered too difficult and sensitive by antenatal care providers (Stotland et al. 2010).

In our study, a substantial proportion of women, more so in the rural hospitals than metro, did not attend the hospital until later than 18 weeks of their pregnancy making them ineligible to participate in the study. This attendance pattern was largely due to the maternity care models (Brock et al. 2014) that encourage community based care during early pregnancy. GHiP could be extended to pregnant women over 18 weeks of gestation to address this issue, however, the program would be less effective as women start gaining weight from second trimester onwards (Carmichael et al. 1997; Overcash et al. 2015). A recent report indicates that approximately half of pregnant women in NSW do not attend antenatal care until at least 14 weeks of gestation (Australian Institute of Health and Welfare 2015). Expanding the GHiP service through encouraging early referrals to GHiP from community based service providers such as general practitioners, practice nurses and early pregnancy services is essential.

A large proportion of eligible women agreed to participate in our study when asked by their midwives but did not follow through by enrolling into GHiP. Some women were possibly reluctant to decline a service offered by their midwife, even when they did not have any interest in participating in GHiP, as has been shown to be the case in other weight related interventions (Atkinson et al. 2013; Knight and Wyatt 2010). Low program uptake and retention rates, similar to our findings, have been reported for different types of weight related interventions for pregnant women in Australia (Chwah et al. 2016; Davis et al. 2012) and internationally (Knight and Wyatt 2010; Poston et al. 2013) that had face to face components. This pattern of participation implies that the telephone-based nature of GHiP is unlikely to have been the main reason for low participation, however, the finding also indicates that not all women prefer a telephone based service or via printed resources only and that other types of services will still be required.

Many pregnant women do not seem to prioritise weight as an issue during pregnancy which is most likely the underlying reason for the low uptake and high withdrawal from the program. Some of the women who suggested lack of time as their reason for withdrawing from the program may have done so due to non-interest as the calls would have taken only 10–15 min of their time. Similar findings of women not prioritising weight during pregnancy have been reported in previous studies (Gaudet et al. 2011; Shub et al. 2013). Another important finding from our study is that the women who were overweight or obese at pre-pregnancy were more likely to withdraw from the program than women with normal pre-pregnancy BMI. This withdrawal is a concern, as overweight and obese women have an increased risk of adverse pregnancy outcomes (Athukorala et al. 2010). These findings suggest that approaches to increase the knowledge and awareness about weight gain during pregnancy are required alongside weight management programs.

Our study was not fully able to assess the effectiveness of GHiP, as originally intended, because of the low level of program uptake and retention and the disproportionally higher withdrawal rates for overweight and obese women. Other limitations were a pure control group was lacking and the pre-pregnancy and post-program weight data was self reported by the women. Recall errors in self-estimated pre-pregnancy weight (McClure et al. 2011) may have led to incorrect classification of women into the normal BMI and overweight or obese categories which would have resulted in those women being given the wrong target range for weight gain. However, this approach to data collection is in line with pragmatic nature of our trial as weight gain advice is given by midwives in NSW based on self-reported pregnancy BMI. Future research should aim to compare higher and lower intensity variations of the service to assess relative impacts. Stronger qualitative data may also help explain how to maximise the intervention effect.

Despite the above limitations, our study suggests that GHiP is a valuable cost-effective addition to routine antenatal care that can be easily incorporated into the current care model for supporting women in NSW to achieve a healthy gestational weight gain, raise awareness of the importance of healthy weight gain in pregnancy and help facilitate conversations between health professionals and pregnant women in line with antenatal care guidelines. Feedback from the women about GHiP was generally positive and indicated a level of acceptance among users.

Telephone based health coaching does not suit all pregnant women which makes the conversations between the health professionals and pregnant women even more important. One of the most important outcomes of integrating the GHiP program with the hospital antenatal services has been the facilitation of the conversations about weight between the pregnant women and their midwives and the medical practitioners. To help pregnant women manage their weight in pregnancy, these type of conversations should be happening not only at the hospital antenatal clinics but also earlier during the pregnancy. Early pregnancy care providers such as GPs and community nurses should discuss weight gain during pregnancy with the pregnant women. Programs such as GHiP can offer an accessible and a cost-effective referral pathway for the women these health practitioners see a need for additional support to manage their weight during pregnancy.

## Data Availability

The datasets generated and/or analysed during the current study are not publicly available as they contain identifiers routinely collected by the midwives and the health coaches. The data that support the findings of this study are also available from Health ways Australia, the external service provider that delivers the GHiP program. However, restrictions apply to the availability of these data, and so are not publicly available. All requests for data will have to be made to the Get Healthy Service Manager at the NSW Office of Preventive Health.
